# Geriatric Nutritional Risk Index Assessment in Patients Undergoing Transcatheter Edge-to-Edge Repair

**DOI:** 10.1016/j.jacadv.2025.101631

**Published:** 2025-02-25

**Authors:** Kenichi Shibata, Masanori Yamamoto, Ai Kagase, Takahiro Tokuda, Hiroshi Tsunamoto, Testuro Shimura, Azusa Kurita, Ryo Yamaguchi, Mike Saji, Masahiko Asami, Yusuke Enta, Masaki Nakashima, Shinichi Shirai, Masaki Izumo, Shingo Mizuno, Yusuke Watanabe, Makoto Amaki, Kazuhisa Kodama, Junichi Yamaguchi, Toru Naganuma, Hiroki Bota, Yohei Ohno, Masahiro Yamawaki, Daisuke Hachinohe, Hiroshi Ueno, Kazuki Mizutani, Toshiaki Otsuka, Shunsuke Kubo, Kentaro Hayashida, the OCEAN-Mitral Investigators

**Affiliations:** a Department of Rehabilitation, Nagoya Heart Center, Nagoya, Japan; b Department of Cardiology, Nagoya Heart Center, Nagoya, Japan; c Department of Cardiology, Toyohashi Heart Center, Toyohashi, Japan; d Department of Cardiology, Gifu Heart Center, Gifu, Japan; e Department of Cardiology, Sakakibara Heart Institute, Fuchu, Japan; f Division of Cardiology, Mitsui Memorial Hospital, Tokyo, Japan; g Department of Cardiology, Sendai Kosei Hospital, Sendai, Japan; h Division of Cardiology, Kokura Memorial Hospital, Kitakyushu, Japan; i Division of Cardiology, St. Marianna University School of Medicine Hospital, Kawasaki, Japan; j Department of Cardiology, Shonan Kamakura General Hospital, Kanagawa, Japan; k Department of Cardiology, Teikyo University School of Medicine, Tokyo, Japan; l Department of Cardiology, National Cerebral and Cardiovascular Center, Suita, Japan; m Division of Cardiology, Saiseikai Kumamoto Hospital Cardiovascular Center, Kumamoto, Japan; n Department of Cardiology, Tokyo Woman’s Medical University, Tokyo, Japan; o Department of Cardiology, New Tokyo Hospital, Chiba, Japan; p Department of Cardiology, Sapporo Higashi Tokushukai Hospital, Sapporo, Japan; q Department of Cardiology, Tokai University School of Medicine, Isehara, Japan; r Department of Cardiology, Saiseikai Yokohama City Eastern Hospital, Kanagawa, Japan; s Department of Cardiology, Sapporo Heart Center, Sapporo Cardio Vascular Clinic, Sapporo, Japan; t Second Department of Internal Medicine, Toyama University Hospital, Toyama, Japan; u Division of Cardiology, Department of Medicine, Kinki University Faculty of Medicine, Osaka, Japan; v Department of Hygiene and Public Health, Nippon Medical School, Tokyo, Japan; w Department of Cardiology, Kurashiki Central Hospital, Kurashiki, Japan; x Department of Cardiology, Keio University School of Medicine, Tokyo, Japan

**Keywords:** Geriatric Nutritional Risk Index, mitral regurgitation, predictor, risk stratification, transcatheter edge-to-edge repair

## Abstract

**Background:**

Transcatheter edge-to-edge repair (TEER) is used to treat patients with mitral regurgitation (MR). The Geriatric Nutritional Risk Index (GNRI) is a well-known nutritional marker that predicts mortality risk.

**Objectives:**

The objectives of this study were to elucidate the clinical association between the degree of GNRI and different etiologies of MR and to clarify the patient samples for whom GNRI is more relevant to clinical outcomes following TEER.

**Methods:**

Data from 3,554 patients with MR who underwent TEER were analyzed using a Japanese multicenter registry. The patients were classified into 4 groups: GNRI <82, GNRI 82 to 92, GNRI 92 to 98, and GNRI >98. Procedural and clinical outcomes were compared between GNRI groups. Short- and long-term all-cause mortality were explored using Cox regression analysis.

**Results:**

Among the 3,554 patients, the median GNRI was 92.3. The mean follow-up period was 586.8 ± 436.5 days; 806 patients died during the follow-up period. Thirty-day mortality occurred in 51 patients (1.4%), and the GNRI <82 group had the highest 30-day mortality rate. Kaplan–Meier curves showed significantly better prognoses for the entire cohort, functional MR, and degenerative MR across the 4 groups (*P* < 0.001). GNRI values, even after adjustment for multiple confounders, showed a stepwise increase in risk of death in the GNRI 92 to 98, GNRI 82 to 92, and GNRI <82 groups compared to GNRI >98 as the reference.

**Conclusions:**

Regardless of MR etiology, GNRI is a useful predictor of short- and long-term mortality in patients undergoing TEER. Although TEER is effective for MR patients in malnourished states, further studies focused on the value of identifying and addressing malnutrition in this population are needed.

Transcatheter edge-to-edge repair (TEER) is a novel therapeutic approach for valvular heart disease with mitral regurgitation (MR). Previous pivotal randomized trials have revealed the effectiveness of TEER using the MitraClip (Abbott Vascular) in patients with both degenerative mitral regurgitation (DMR) and functional mitral regurgitation (FMR).^1^^,^^2^ With the widespread use of TEER in daily practice, careful patient screening and risk assessment are required before invasive therapy.

Malnutrition is a key factor in the progression of frailty and is associated with an increased risk of mortality.^3^^,^^4^ A well-known simple marker, the Geriatric Nutritional Risk Index (GNRI) has been proposed and investigated in the prediction of risk of nutrition-related complications and increased risk of mortality among the elderly sample.^5^ Furthermore, the subanalysis of COAPT (Cardiovascular Outcomes Assessment of the MitraClip Percutaneous Therapy for Heart Failure Patients with Functional Mitral Regurgitation) trial identifies the significant relationship between the baseline malnutrition status defined by GNRI ≤98 and increased risk of mortality, but not heart failure rehospitalization (HFH), regardless of patients receiving TEER or not.^6^ However, the results of the subanalysis focused on the FMR cohort under strict eligibility criteria and consisted of a small number of patients with moderate/severe malnutrition (GNRI ≤92/<82) status. The clinical impact of GNRI is unclear in DMR patients after TEER. Patients with FMR are considered as a subset of heart failure (HF) with mixture of left atrial damage, reduced left atrium and/or heart cardiac function, whereas patients with DMR have mitral valve disease with impairment of the mitral valve itself. Therefore, the baseline characteristics and therapeutic approaches differ in patients with FMR and DMR. Thus, additional nutritional information is required for patients with FMR and DMR, severely impaired nutritional status, and nonselected real-world samples. We, therefore, aimed to elucidate the association between nutritional status stratified by GNRI and clinical outcomes, including HFH after TEER stratified by FMR and DMR, and to clarify patient samples for whom nutritional assessment is more relevant to clinical outcomes in TEER patients.

## Methods

### Patient sample and study design

OCEAN-Mitral is an ongoing, prospective, investigator-initiated, multicenter registry that assesses the safety and efficacy of TEER in patients with significant MR.^7^^,^^8^ A total of 21 Japanese institutions participated in this registry. Between April 2018 and June 2023, 3,764 consecutive patients with symptomatic MR underwent TEER. The GNRI is a tool that provides an accepted clinical definition of malnutrition.^5^^,^^9^ In the entire cohort, we lacked 25 GNRI data points (missing information: height, n = 1; weight, n = 1; albumin, n = 23), as well as 185 patients who did not achieve TEER acute procedural success, defined as maintaining procedural safety without life-threatening complications and adequate reduction of MR at discharge. A total of 3,554 patients were included in this study.

The method for evaluation of GNRI has been reported (GNRI = 1.489 × albumin [g/l] + 41.7 × body weight [kg]/ideal body weight [kg]) and, as in previous studies, in patients with preprocedural body weight greater than ideal body weight, we set body weight/ideal body weight ratio = 1.^5^^,^^10^ The patients were divided into the following 4 groups based on the report of Bouillanne et al^5^: GNRI <82 (n = 590, 16.6%), GNRI 82 to 92 (n = 1,093, 30.8%), GNRI 92 to 98 (n = 849, 23.9%), and GNRI >98 (n = 1,022, 28.8%). The prognostic value of the GNRI was assessed for the entire cohort, the FMR, and the DMR.

This study was registered in the University Hospital Medical Information Network Clinical Trials Registry with the International Committee of Medical Journal Editors (UMIN000023653). All patients provided informed consent before undergoing the TEER procedure, and the study protocol was approved by the institutional review board of each institution. This study was conducted in accordance with the latest version of the Declaration of Helsinki and the guidelines for epidemiological studies issued by the Ministry of Health, Labor, and Welfare of Japan.

### TEER procedure

The MitraClip (Abbott Vascular) is the only commercially available mitral transcatheter device in Japan. After the introduction of the MitraClip Generation 2 (G2) system, the latest version of the G4 system (excluding G3) was launched in September 2020. The MitraClip G4 system has 4 different size variations (NT, NTW, XT, and XTW) that aim to adapt to the different mitral valve morphologies of individual patients. TEER is indicated in patients with moderate-to-severe MR regardless of the MR etiology. Baseline MR severity was assessed based on the guidelines of the American Society of Echocardiography,^11^ and after the TEER procedure, as previously described.^12^

MR severity was classified as 0+ (none/trivial), 1+ (mild), 2+ (moderate), 3+ (moderate-to-severe), and 4+ (severe). Individual heart team members considered the appropriateness of TEER based on patient background, including age, frailty, and operative risk of cardiac surgery. The detailed TEER procedure has been previously reported.^7^^,^^8^ All TEER procedures were performed under general anesthesia and transesophageal echocardiography guidance. An acceptable MR reduction after clipping the mitral valve was defined as MR ≤2+ using perioperative transesophageal echocardiography findings. If further MR reduction was required, the physicians attempted to change the position of the first clip or implant a second or third clip depending on the situation.

### Clinical outcome measures and definitions

Baseline characteristics, laboratory data, transthoracic echocardiography findings, and transesophageal echocardiography findings were examined at each center. Clinical follow-up was conducted at baseline and 1, 12, and 24 months after TEER. At each visit, patients were asked about their history of HFH after TEER. If patients could not visit the hospital, clinical data, including death information, were collected through telephone interviews with the patients, their family members, or their relatives. The cause of death was divided into cardiac and noncardiac profile based on the definition of the Mitral Valve Academic Research Consortium.^13^ In brief, cardiac death includes HF, myocardial infarction, stroke, cardiac arrhythmia, unknown cause including (sudden death), and device-related death. The baseline patient data, procedural variables, and clinical outcomes were compared between the FMR and DMR subgroups. In addition, the baseline GNRI categories and clinical outcomes after TEER were evaluated. The primary endpoints of this study were the incidences of short- and long-term all-cause mortality and HFH after TEER.

### Statistical analysis

Data are presented as mean ± SD or median for continuous variables, and as frequencies (percentages) for categorical variables, unless otherwise indicated. Data comparisons among the groups were made using chi-square tests for categorical covariates and 1-way analysis of variance for continuous covariates. Statistical significance was set at *P* < 0.05, and 95% CIs were reported as appropriate. Cox regression models were constructed to assess the association between GNRI and 30-day mortality. Adjustment of the models included age, sex, and clinical variables with *P* < 0.10 in the univariable analysis, with Model 1 inputs including GNRI as a continuous variable, and model 2 with GNRI imputed categorically into 4 groups according to the previous formula. The Kaplan–Meier method for unadjusted models was used to estimate the cumulative incidence, and differences in mortality and HFH assessed using the log-rank test. We also used the cumulative incidence function to estimate the cumulative incidence of HFH, accounting for competing risk, with death without HFH treated as a competing event. The Gray model was developed to analyze the association with the risk of HFH while appropriately addressing competing risks. A univariable Cox regression analysis was performed to obtain the HRs for long-term mortality during the follow-up period. A multivariable analysis was performed to detect the independent predictors of death, and covariates with *P* < 0.10 in the univariable analysis were included in the multivariable model. Similarly, HFH was considered a competing risk and HRs were calculated using Fine and Gray analysis. Potential for effect modification was assessed by survival analysis in subgroups in body mass index (BMI) (stratified by 20 and 25 kg/m^2^) and B-type natriuretic peptide (BNP) values (stratified by median). Improvements in the predictive accuracy of GNRI compared with the conventional prediction of survival using the clinical model and basic components were determined by calculating the net reclassification improvement (NRI) and integrated discrimination improvement (IDI) based on a logit model.^14^ All statistical analyses were performed using EZR (Saitama Medical Center, Jichi Medical University, Saitama, Japan), a graphical user interface for R (R Foundation for Statistical Computing).^15^

## Results

### Baseline characteristics and procedural variables

The baseline patient characteristics are shown in Table 1. Several clinical characteristics differed among the 4 groups, including age, sex, body characteristics, CFS, prior HFH, prevalence of NYHA class III/IV, hypertension, dyslipidemia, diabetes mellitus, chronic kidney disease, and peripheral artery disease (all *P* < 0.05). Echocardiographic parameters such as left ventricular ejection fraction, left ventricular end-diastolic diameter, and end-systolic diameter also showed signiﬁcant differences between the 4 groups (all *P* < 0.05).

**Table 1:** Baseline Characteristics of Study Participants

	GNRI <82 (n = 590)	GNRI 82-92 (n = 1,093)	GNRI 92-98 (n = 849)	GNRI >98 (n = 1,022)	*P* Value
Baseline clinical characteristics					
Age, y	81.2 ± 8.7	79.5 ± 9.6	79.0 ± 8.9	76.1 ± 10.1	<0.001
≥80 y	381 (64.6%)	646 (59.1%)	500 (58.9%)	454 (44.4%)	<0.001
Male	273 (46.3%)	570 (52.2%)	486 (57.2%)	622 (60.9%)	<0.001
Body height, cm	155.3 ± 10.0	156.3 ± 10.5	157.3 ± 10.0	158.3 ± 9.9	<0.001
Body weight, kg	45.5 ± 10.5	49.7 ± 10.3	53.9 ± 10.5	59.0 ± 10.7	<0.001
BMI, kg/m^2^	18.7 ± 3.2	20.3 ± 3.0	21.7 ± 3.1	23.4 ± 3.1	<0.001
Heart rate, beats/min	78.4 ± 16.6	75.3 ± 16.0	73.4 ± 15.1	72.3 ± 14.4	<0.001
Functional MR	423 (71.7%)	785 (71.8%)	616 (72.6%)	701 (68.6%)	0.224
Clinical frailty scale, points	4 (4-6)	4 (3-5)	4 (3-4)	3 (3-4)	<0.001
STS score for mitral valve replacement, (%, n = 3,350)	12.4 (8.2-19.5)	9.9 (6.4-15.0)	8.7 (5.7-12.4)	6.6 (4.3-10.1)	<0.001
Prior HFH within 12 mo prior to enrollment	504 (86.0%)	852 (78.4%)	590 (70.0%)	637 (62.4%)	<0.001
NYHA class, III or IV	472 (80.0%)	753 (68.9%)	461 (54.3%)	549 (53.7%)	<0.001
Preprocedural nutritional status and laboratory data					
GNRI	76.3 ± 4.7	87.4 ± 2.7	94.8 ± 1.7	102.7 ± 3.8	<0.001
Albumin, g/dL	2.8 ± 0.4	3.4 ± 0.3	3.8 ± 0.3	4.2 ± 0.3	<0.001
BNP, pg/mL (n = 2,579)	560.7 (267.7-1,132.6)	431.0 (216.2-816.1)	323.5 (164.3-614.2)	236.8 (124.2-445.9)	<0.001
Estimated glomerular filtration rate, mL/min/1.73 m^2^	38.4 ± 23.4	37.3 ± 18.4	39.2 ± 18.5	41.6 ± 18.2	<0.001
<30 mL/min/1.73 m^2^	243 (41.4%)	379 (34.9%)	282 (33.3%)	277 (27.2%)	<0.001
Hemoglobin, g/dL	10.6 ± 1.6	11.3 ± 1.6	11.9 ± 1.7	12.7 ± 1.8	<0.001
High-sensitivity CRP, mg/dL (n = 3,325)	0.80 (0.21-2.48)	0.25 (0.09-0.78)	0.12 (0.05-0.31)	0.10 (0.05-0.21)	<0.001
Comorbidities					
Hypertension	366 (62.0%)	686 (62.8%)	560 (66.0%)	706 (69.1%)	0.006
Dyslipidemia	254 (43.1%)	493 (45.1%)	418 (49.2%)	559 (54.7%)	<0.001
Diabetes mellitus	136 (23.1%)	275 (25.2%)	237 (27.9%)	293 (28.7%)	0.047
Chronic kidney disease	494 (83.7%)	968 (88.6%)	737 (86.8%)	881 (86.2%)	0.046
Dialysis dependent	63 (10.7%)	81 (7.4%)	38 (4.5%)	33 (3.2%)	<0.001
Atrial fibrillation/flutter	370 (62.7%)	676 (61.8%)	533 (62.8%)	605 (59.2%)	0.349
Prior stroke	71 (12.0%)	127 (11.6%)	92 (10.8%)	119 (11.6%)	0.904
Liver cirrhosis	11 (1.9%)	17 (1.6%)	13 (1.5%)	18 (1.8%)	0.945
Chronic obstructive lung disease	45 (7.6%)	98 (9.0%)	70 (8.2%)	88 (8.6%)	0.809
Peripheral artery disease	70 (11.9%)	118 (10.8%)	86 (10.1%)	74 (7.2%)	0.008
Echocardiographic data					
LV ejection fraction, %	43.9 ± 16.5	43.8 ± 16.5	45.8 ± 16.3	45.7 ± 16.8	0.006
<40%	277 (46.9%)	519 (47.6%)	357 (42.1%)	459 (44.9%)	0.091
LV end-diastolic diameter, mm	53.8 ± 10.5	56.1 ± 9.9	56.9 ± 10.3	58.6 ± 10.5	<0.001
LV end-systolic diameter, mm	42.1 ± 13.0	43.9 ± 12.9	44.0 ± 13.6	45.3 ± 14.0	<0.001
LA volume index, cm^3^/m^2^	83.5 ± 43.7	85.9 ± 45.9	88.6 ± 51.3	86.6 ± 56.2	0.33
MR characteristics					
Moderate-severe MR	583 (98.8%)	1,078 (98.6%)	1,835 (98.4%)	1,001 (97.9%)	0.503
MR PISA EROA, cm^2^	0.39 ± 0.21	0.38 ± 0.21	0.38 ± 0.18	0.37 ± 0.18	0.064
MR regurgitation volume, mL	53.8 ± 24.5	54.9 ± 24.2	57.8 ± 39.3	55.0 ± 30.6	0.079
TMPG, mm Hg	1.97 ± 1.10	1.90 ± 1.09	1.86 ± 1.20	1.79 ± 0.99	0.018

Values are mean ± SD, n (%), or median (IQR).

BMI = body mass index; BNP = B-type natriuretic peptide; CRP = C-reactive protein; EROA = effective regurgitant orifice area; HFH = heart failure hospitalization; LV = left ventricular; MR = mitral regurgitation; PISA = proximal isovelocity surface area; STS = Society of Thoracic Surgeons Predictive Risk of Mortality; TMPG = transmitral mean pressure gradient.

The procedural variables are presented in Table 2. The proportion of MR ≥2+ and procedure times did not differ significantly among the 4 groups; however, intensive care unit and hospital stays were prolonged across the GNRI >98, GNRI 92 to 98, GNRI 82 to 92, and GNRI <82 groups. The rates of procedural complications such as acute kidney injury showed signiﬁcant differences between the 4 groups (*P* < 0.05). In-hospital mortality was markedly higher in the GNRI <82 group (GNRI<82: 10.5%, GNRI 82-92: 1.6%, GNRI 92-98: 0.5%, GNRI >98: 0.4%, respectively, *P* < 0.001). Additionally, baseline patient characteristics and procedural variables by etiology of MR are shown in Supplemental Table 1. Compared to the DMR group, the FMR group was younger, had a higher proportion of males, and had a higher history of HFH within 12 months prior to enrollment. The FMR group also had higher BNP levels, lower left ventricular ejection fraction, and larger left ventricular diameter.

**Table 2:** Procedural Results

	GNRI <82 (n = 590)	GNRI 82-92 (n = 1,093)	GNRI 92-98 (n = 849)	GNRI >98 (n = 1,022)	*P* Value
Procedural variables					
Post MR ≥ 2+	67 (11.4)	133 (12.2)	120 (14.2)	145 (14.2)	0.24
Procedural time, min	88.3 ± 41.8	87.9 ± 43.6	90.6 ± 43.2	90.6 ± 44.8	0.433
Device time, min	59.8 ± 34.5	61.3 ± 37.1	63.1 ± 36.2	62.5 ± 34.3	0.424
Fluoroscopy time, min	26.8 ± 15.0	27.5 ± 18.4	29.2 ± 20.0	29.5 ± 19.5	0.017
Length of hospital stay, d	37.3 ± 38.5	24.1 ± 26.6	17.0 ± 18.3	15.4 ± 14.9	<0.001
Length of ICU stay, d	4.7 ± 10.2	2.1 ± 5.3	1.5 ± 2.9	1.3 ± 1.5	<0.001
MitraClip detail information					
Number of implanted clips	1.24 ± 0.45	1.25 ± 0.46	1.30 ± 0.48	1.30 ± 0.48	0.014
G2 NT	275 (37.5)	596 (43.5)	525 (47.5)	649 (48.9)	
G4 NT	102 (13.9)	190 (13.9)	128 (11.6)	160 (12.1)	
G4 NTW	192 (26.1)	306 (22.4)	223 (20.2)	242 (18.2)	
G4 XT	27 (3.7)	47 (3.4)	49 (4.4)	59 (4.4)	
G4 XTW	138 (18.8)	230 (16.8)	180 (16.3)	218 (16.4)	
Procedural complications and outcomes					
Access site related complications	9 (1.5)	15 (1.4)	16 (1.9)	17 (1.7)	0.837
Acute kidney injury (AKIN stage 2 or 3)	23 (3.9)	20 (1.8)	11 (1.3)	13 (1.3)	0.001
Pulmonary complications	4 (0.7)	10 (0.9)	3 (0.4)	2 (0.2)	0.115
TEE associated complications	4 (0.7)	11 (1.0)	6 (0.7)	6 (0.6)	0.713
In-hospital death	62 (10.5)	18 (1.6)	4 (0.5)	4 (0.4)	<0.001

Values are n (%) or mean ± SD.

AKIN = Acute Kidney Injury Network; G2 = generation 2; G4 = generation 4; ICU = intensive care unit; MR = mitral regurgitation; TEE = transesophageal echocardiography.

### GNRI and short- and long-term all-cause mortality

**Figure 1:**
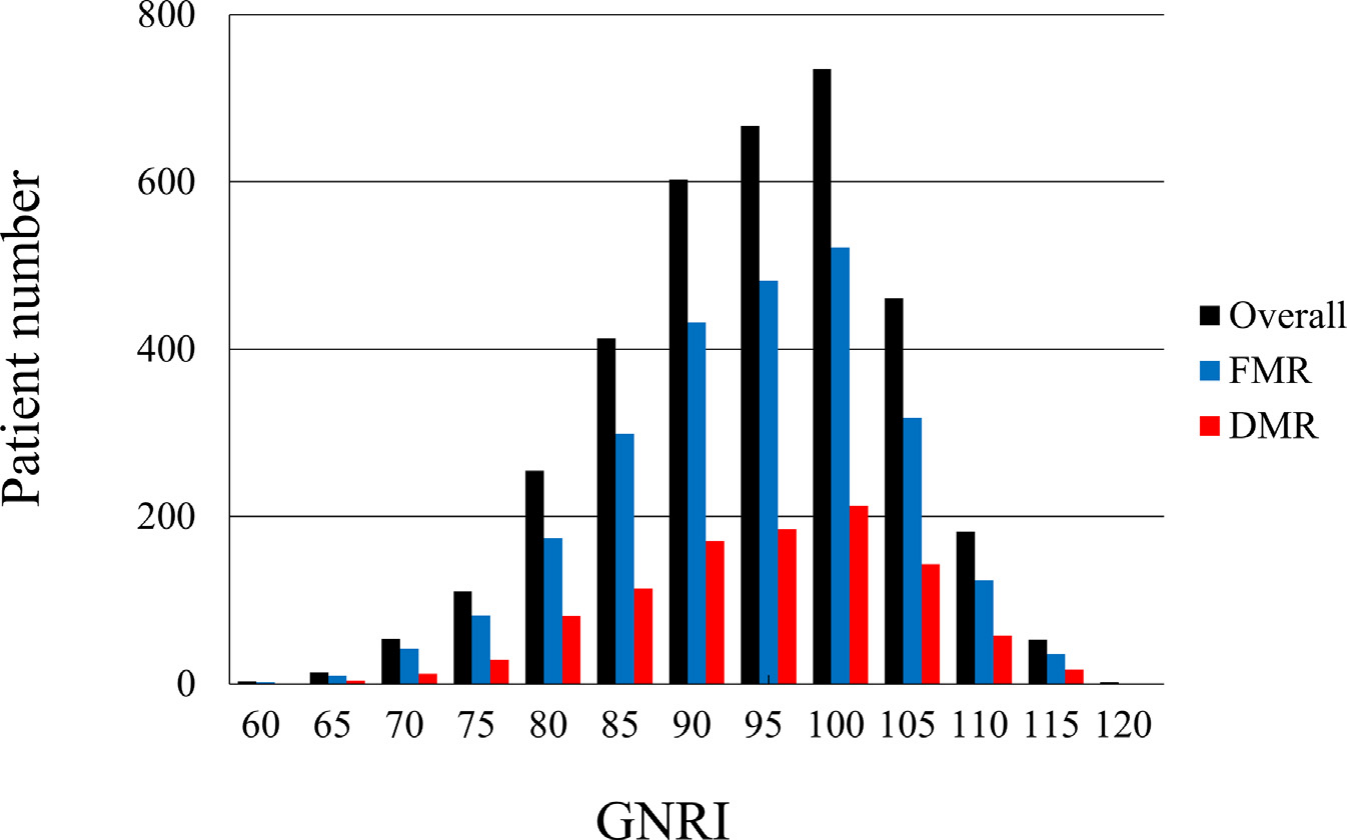
Preprocedural GNRI Distribution in Overall, FMR, and DMR Samples

**Figure 2:**
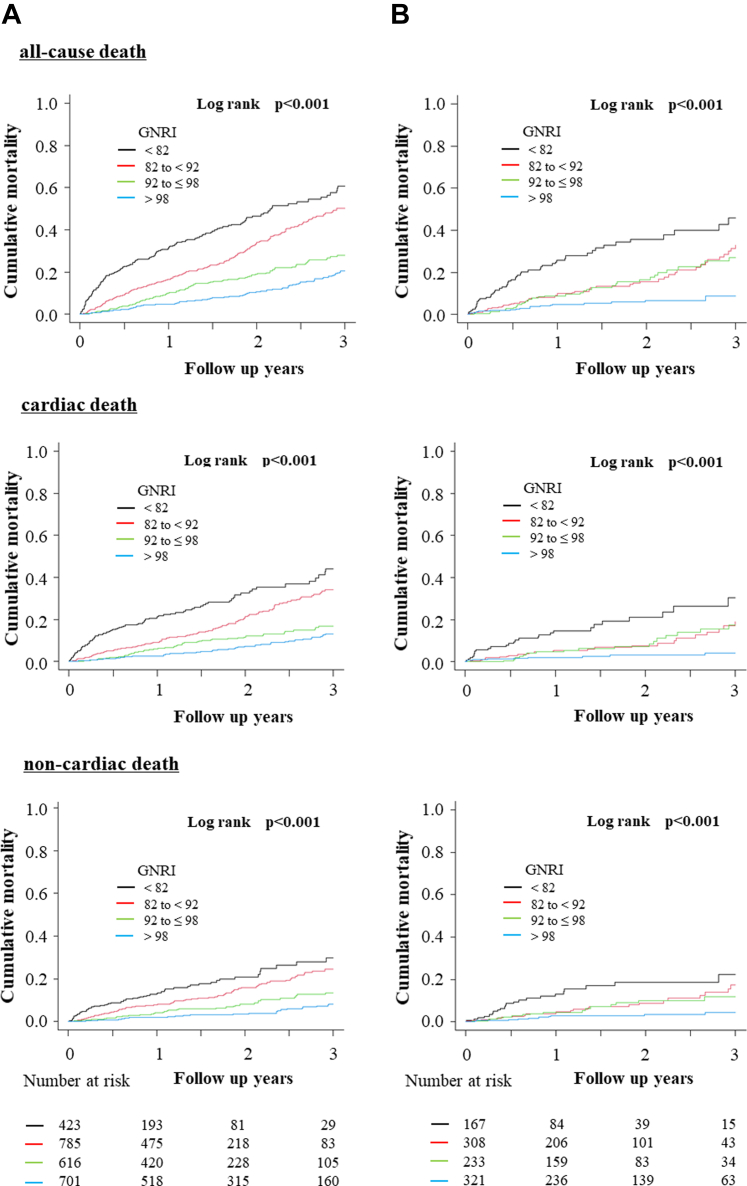
Kaplan–Meier All-Cause, Cardiac and Noncardiac Mortality According to GNRI Kaplan–Meier curve of all-cause, cardiac and non-cardiac mortality in functional mitral regurgitation (FMR) (A), and in degenerative mitral regurgitation (DMR) (B).

The distribution of the GNRI in the overall, FMR, and DMR groups is shown in Figure 1. Among the 3,554 patients, the median GNRI was 92.3 (IQR: 85.4-98.4; minimum, 55.0; maximum, 120.6). The mean follow-up period was 586.8 ± 436.5 days for the entire cohort, and 806 patients (22.7%; FMR: 632 patients, DMR: 174 patients) died during the follow-up period. Thirty-day mortality occurred in 51 patients (1.4%), and the GNRI <82 group had the highest 30-day mortality rate (GNRI <82: 5.6%, GNRI 82-92: 1.0%, GNRI 92-98: 0.4%, GNRI >98: 0.4%, respectively, *P* < 0.001). The cutoff value of the GNRI from the receiver operating characteristic curve for 30-day mortality was 84.7 (sensitivity, 0.780; specificity, 0.745; area under the curve, 0.801). In the multivariable Cox regression analysis, GNRI <82 group was significantly associated with 30-day all-cause mortality compared with GNRI >98 as a reference (HR: 5.41 [95% CI: 1.72-17.03, *P* = 0.004]) (Table 3). The Kaplan–Meier curves indicated signiﬁcant differences in all-cause, cardiovascular, and noncardiovascular mortality up to 3 years between the 4 groups (Central Illustration). Patients in the higher GNRI group had a significantly better prognosis for the FMR and DMR (log-rank test, all *P* < 0.001) (Figure 2).

**Table 3:** Univariable and Multivariable Cox Regression Analyses for 30-Day All-Cause Mortality

	Univariable Analysis			Multivariable Analysis					
	HR	95% CI	*P* Value	HR	95% CI	*P* Value	HR	95% CI	*P* Value
Adjusting factors					Model 1			Model 2	
GNRI (per 1 scale increase)	0.89	0.87-0.91	<0.001	0.92	0.89-0.95	<0.001			
GNRI >98	reference						reference		
GNRI 92-≤98	0.90	0.20-4.03	0.89				0.67	0.15-3.02	0.60
GNRI 82-<92	2.58	0.82-8.10	0.10				1.26	0.38-4.19	0.71
GNRI <82	14.77	5.23-41.7	<0.001				5.41	1.72-17.03	0.004
Age (per 1 y increase)	1.02	0.99-1.06	0.13	1.03	0.99-1.07	0.16	1.02	0.99-1.06	0.18
Male	1.27	0.73-2.24	0.40	1.93	1.04-3.58	0.037	2.00	1.08-3.71	0.027
Body mass index (per 1 kg/m^2^ increase)	1.00	0.92-1.08	0.99						
NYHA functional class III or IV	3.74	1.69-8.31	0.001	2.09	0.86-5.07	0.10	1.96	0.81-4.77	0.14
Clinical frailty scale (per 1 point increase)	1.62	1.36-1.93	<0.001	1.19	0.98-1.44	0.086	1.23	1.02-1.49	0.031
Hypertension	0.90	0.51-1.58	0.71						
Diabetes mellitus	0.95	0.51-1.78	0.87						
Atrial fibrillation	1.26	0.70-2.25	0.44						
Prior stroke	1.45	0.68-3.07	0.34						
Chronic obstructive pulmonary disease	0.67	0.21-2.15	0.50						
DMR	0.67	0.34-1.3	0.24						
LVEF ≥40%	0.58	0.33-1.01	0.053	0.56	0.30-1.02	0.06	0.56	0.30-1.03	0.064
Hemoglobin (per 1 g/dL increase)	0.64	0.54-0.76	<0.001	0.85	0.70-1.03	0.098	0.82	0.68-1.00	0.047
eGFR (per 1 mL/min/1.73 m^2^ increase)	0.98	0.96-1.00	0.015	0.99	0.97-1.00	0.12	0.99	0.97-1.00	0.11

DMR = degenerative mitral regurgitation; eGFR = estimated glomerular filtration rate; LVEF = left ventricle ejection fraction.

**Central Illustration:**
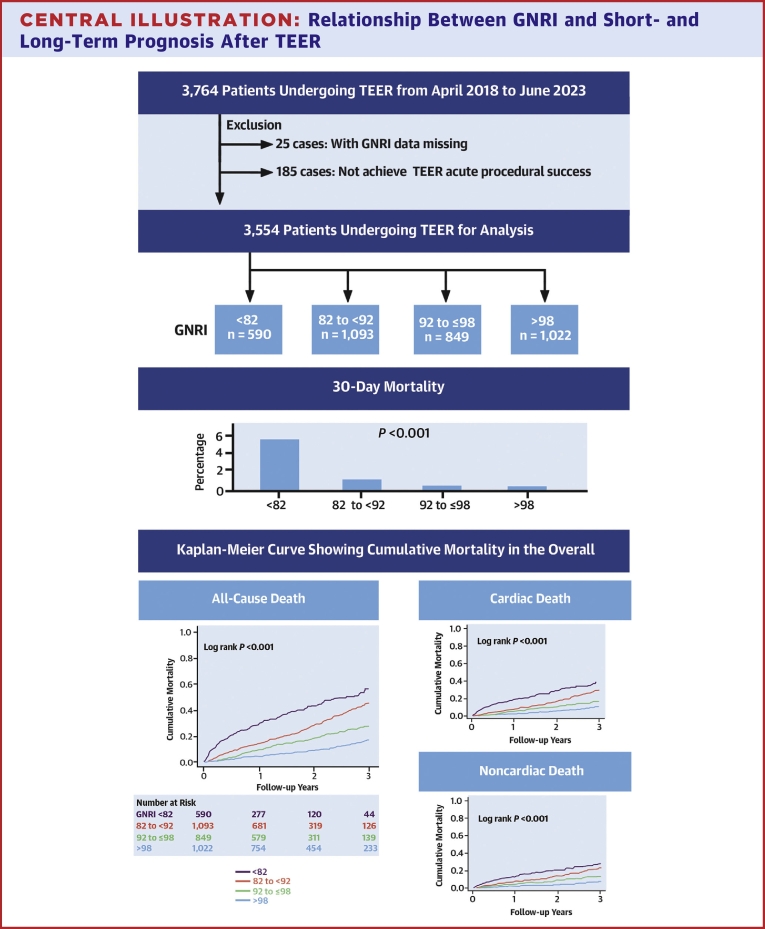
Relationship Between GNRI and Short- and Long-Term Prognosis After TEER

The results of the Cox regression analysis are presented in Table 4. In the univariable Cox regression, GNRI values demonstrated a stepwise incremental increase in the risk of mortality in the GNRI 92 to 98, GNRI 82 to 92, and GNRI <82 groups compared with GNRI >98 as a reference. These results were not attenuated, even after adjustment for multiple confounding factors (for GNRI 92-98: HR, 1.24 [95% CI, 0.92-1.66, *P* = 0.16]; GNRI 82 to <92: HR, 1.89 [95% CI, 1.44-2.47, *P* < 0.001]; GNRI <82: HR, 2.61 [95% CI, 1.92-3.56, *P* < 0.001], respectively). This trend was similar in the FMR group, although only GNRI 92 to 98 was not significant (HR, 1.02 [95% CI, 0.73-1.43, *P* = 0.89]). In the DMR group, a lower GNRI was associated with a significantly worse prognosis than that in the GNRI >98 group. Cox regression of cardiovascular and noncardiovascular deaths showed similar results, with a significantly increased risk of death based with lower GNRI, even after adjustment for multiple confounding factors, regardless of differences in MR etiology (Supplemental Table 2).

**Table 4:** Univariable and Multivariable Cox Regression Analyses for All-Cause Mortality

	Overall			FMR			DMR		
	HR	95% CI	*P* Value	HR	95% CI	*P* Value	HR	95% CI	*P* Value
Univariable analysis									
GNRI >98	Reference			Reference			Reference		
GNRI 92-≤98	1.78	1.40-2.25	<0.001	1.52	1.17-1.99	0.002	2.92	1.75-4.89	<0.001
GNRI 82-<92	2.97	2.41-3.67	<0.001	2.93	2.32-3.71	<0.001	3.18	1.94-5.19	<0.001
GNRI <82	5.40	4.32-6.74	<0.001	5.06	3.95-6.50	<0.001	6.97	4.22-11.54	<0.001
Multivariable analysis									
GNRI >98	Reference			Reference			Reference		
GNRI 92-≤98	1.24	0.92-1.66	0.16	1.02	0.73-1.43	0.89	2.91	1.47-5.78	0.002
GNRI 82-<92	1.89	1.44-2.47	<0.001	1.67	1.24-2.25	<0.001	3.34	1.72-6.49	<0.001
GNRI <82	2.61	1.92-3.56	<0.001	2.22	1.58-3.13	<0.001	5.94	2.80-12.61	<0.001

Multivariable models were adjusted for: age, sex, hemoglobin, prior HFH within 12 months prior to enrollment, New York Heart Association functional class III or IV, heart rate, atrial fibrillation/flutter, eGFR, clinical frailty scale, LVEF <40%, diabetes mellitus, BNP, dialysis dependent, Peripheral artery disease, functional MR (Overall only).

BNP = B-type natriuretic peptide; eGFR = estimated glomerular filtration rate; HFH = heart failure hospitalization; LVEF = left ventricle ejection fraction.

The results of the NRI and IDI analyses of the incremental value of the GNRI when added to the predictive model and basic components are presented in Supplemental Table 3. The NRI and IDI showed significant improvements for predicting mortality when combining GNRI and clinical model consisting of age, sex, and other independent predictors of death (NRI: 0.292; 95% CI: 0.198-0.385, *P* < 0.001; IDI: 0.017, 95% CI: 0.011-0.023, *P* < 0.001). Moreover, the predictive model consisting of the clinical model and GNRI was superior to the model consisting of the clinical model and each of the other basic components.

### GNRI and HFH

**Figure 3:**
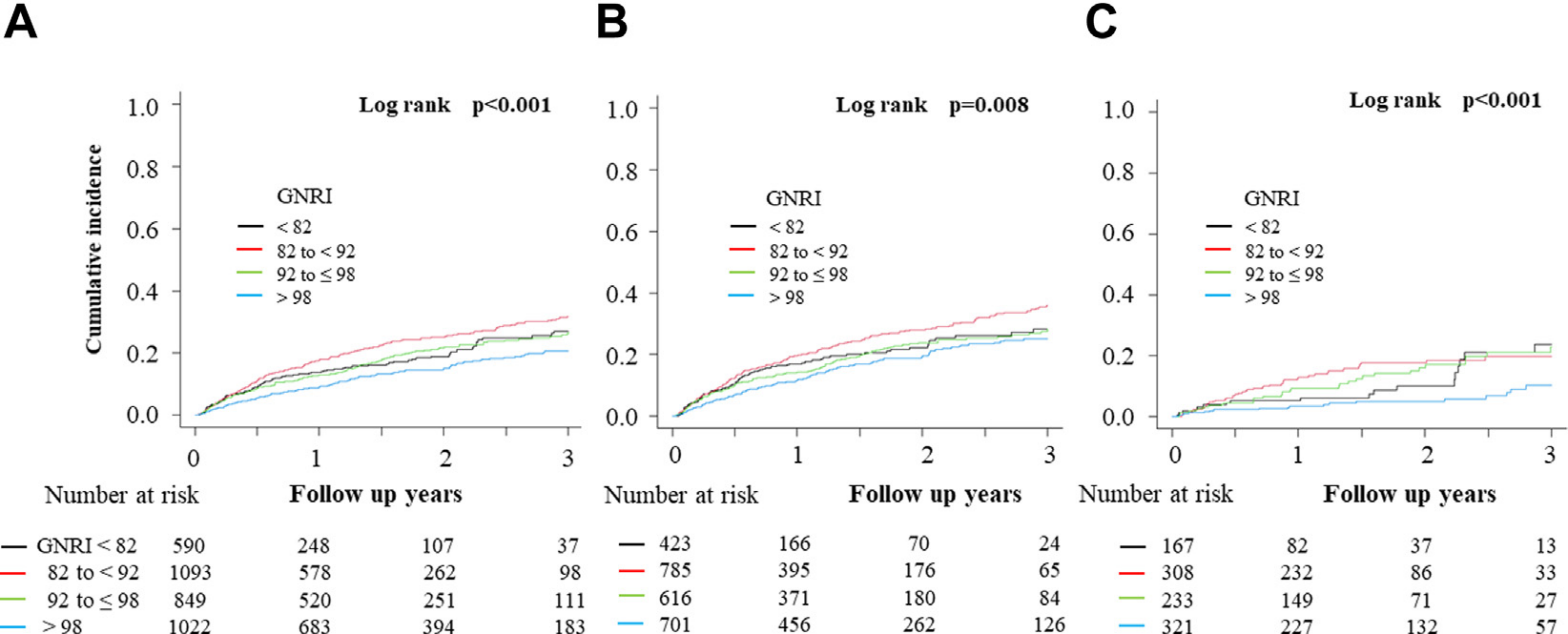
Kaplan–Meier Competing Risk for HFH According to GNRI Kaplan–Meier curve of competing risk for HFH in overall (A), in functional mitral regurgitation (FMR) (B), and in degenerative mitral regurgitation (DMR) (C).

The mean follow-up period was 518.9 ± 421.6 days, and 668 patients (18.8%; FMR, 547; DMR, 121) underwent HFH. Kaplan–Meier survival curves of competing risk for HFH compared to GNRI are shown in Figure 3. Like all-cause mortality, patients in the higher GNRI group had significantly better prognoses in the overall cohort, FMR, and DMR (log-rank test, *P* < 0.001, *P* = 0.008, *P* < 0.001, respectively). Univariable Fine and Gray regression analysis of the association between GNRI and competing risk for HFH also showed an increased risk of rehospitalization in the lower GNRI group than in the GNRI >98 group, except for the FMR patients in the GNRI 92 to 98 and GNRI <82 groups. After adjustment for confounding factors in multivariable analysis, only the GNRI 82 to 92 group had a significantly higher risk of rehospitalization in the entire cohort compared with the GNRI >98 group (HR, 1.37 [95% CI: 1.03-1.83, *P* = 0.028]). There was no significant difference in the risk of rehospitalization in any of the GNRI categories in the FMR and DMR groups (Table 5).

**Table 5:** Fine and Gray Competing Risk Regression Analysis for HFH

	Overall			FMR			DMR		
	HR	95% CI	*P* Value	HR	95% CI	*P* Value	HR	95% CI	*P* Value
Univariable analysis									
GNRI >98	Reference			Reference			Reference		
GNRI 92-≤98	1.38	1.12-1.72	0.003	1.19	0.94-1.5	0.16	2.59	1.5-4.46	<0.001
GNRI 82-<92	1.65	1.35-2.01	<0.001	1.45	1.17-1.8	<0.001	2.81	1.67-4.73	<0.001
GNRI <82	1.31	1.02-1.67	0.035	1.17	0.89-1.53	0.27	2.11	1.14-3.92	0.018
Multivariable analysis									
GNRI >98	Reference			Reference			Reference		
GNRI 92-≤98	1.16	0.87-1.56	0.32	1.05	0.76-1.45	0.78	1.66	0.83-3.3	0.15
GNRI 82-<92	1.37	1.03-1.83	0.028	1.31	0.95-1.79	0.1	1.72	0.88-3.38	0.11
GNRI <82	1.00	0.68-1.47	1.0	0.93	0.6-1.43	0.73	1.32	0.56-3.14	0.53

Multivariable models were adjusted for: age, sex, hemoglobin, prior HFH within 12 months prior to enrollment, NYHA functional class III or IV, heart rate, atrial fibrillation/flutter, eGFR, clinical frailty scale, LVEF <40%, diabetes mellitus, BNP, Dialysis dependent, peripheral artery disease, functional MR (Overall only).

BNP = B-type natriuretic peptide; eGFR = estimated glomerular filtration rate; HFH = heart failure hospitalization; LVEF = left ventricle ejection fraction.

### Associations of GNRI with all-cause mortality and HFH in the subgroups

A lower GNRI was consistently associated with worse survival, even when stratified by BMI of 20 and 25 kg/m^2^, and by median preprocedural BNP level (log-rank test, all *P* < 0.001). In competing risk analysis for HFH, patients in the higher GNRI group also had a significantly better prognosis when stratified by BMI and BNP level, except for the high BNP subgroup, but not as clearly as in all-cause mortality (log-rank test, all *P* < 0.05) (Supplemental Figures 1 and 2).

## Discussion

This study had 3 important findings. First, the 30-day mortality after TEER was markedly higher in the group with a GNRI <82, indicating that GNRI is a useful predictor of perioperative mortality. Second, GNRI was significantly associated with long-term all-cause (cardiac and noncardiac) mortality after TEER in both the FMR and DMR groups. Third, although a lower GNRI was associated with an increased risk of HFH, after adjusting for confounders, the GNRI categories were not significantly different in the FMR and DMR groups.

The current study was conducted in a small Asian Japanese sample with a small body size, the distribution of GNRI values is considered to be relatively lower than western cohort. In fact, GNRI values in FMR patients in this study are also distributed lower than in the subanalysis of COAPT sample.^6^ Therefore, the clinical results with the current threshold using GNRI values should be carefully evaluated. However, the lowest GNRI <82 group had a significantly higher in-hospital mortality rate (10.5%) than the other groups. Notably, the length of hospital stay lengthened with decreasing GNRI, with the GNRI <82 group averaging 37 days, much longer than the other cohorts.^16^ It is considered the next challenge to find appropriate solutions for patients in a state of malnutrition who have undergone TEER.

The GNRI has been used as a tool to predict the risk of nutrition-related complications in hospitalized elderly patients,^5^ and meta-analyses have shown an association with prognosis in patients with HF.^17^^,^^18^ Previous reports on GNRI and long-term prognosis after TEER have been limited to FMR patients;^6^^,^^19^ however, this study shows that GNRI is a factor for poor long-term prognosis in DMR patients as well as FMR.

There are several possible mechanisms underlying the relationship between the GNRI and all-cause mortality. The GNRI is defined by serum albumin and BMI, with lower values of either index resulting in a lower GNRI. Hypoalbuminemia has been reported to be associated with poor prognosis after TAVR^20^ and in the HF cohort.^21^ The NRI-IDI analysis demonstrated that the prognostic predictive capability of GNRI is superior to its individual components, including albumin and body weight, and even BMI, a widely used prognostic stratification indicator. The American Society for Parenteral and Enteral Nutrition recently reported that inflammation has a significant impact on albumin levels,^22^^,^^23^ and high-sensitivity C-reactive protein and BNP levels were higher in the lower GNRI group in this study, which may reflect the progression of HF, a chronic inflammatory disease. The GNRI may be more accurate in predicting prognosis than serum albumin or BMI alone by reflecting systemic health status due to pathological conditions and weight changes. In addition, in a subanalysis of BMI 20 to 25 kg/m^2^ and BMI>25 kg/m^2^ aimed to correct for differences in body size between Asians and Westerners, patients with low GNRI still had poorer long-term prognosis. The worse prognosis of lower GNRI subset were also similarly found in both low-BNP and high-BNP group.

The association between the GNRI and competing risk for HFH after TEER was not as clear as the association with all-cause mortality, which supports the results of the subanalysis of the COAPT trial.^6^ Similar trends have been observed not only in FMR patients with high-risk factors for HF development, such as reduced cardiac function, left ventricular hypertrophy, and elevated BNP levels, but also in DMR patients, suggesting that GNRI alone is difficult to predict for HFH after TEER, regardless of MR etiology.

The GNRI is a simple and highly practical index that can be calculated using physical measurements and serum albumin levels. A key finding is that the COAPT trial has proven the superiority of TEER over medical therapy in FMR patients, irrespective of their GNRI values. In this context, it should be emphasized that TEER should be performed even in populations with low GNRI values, who are expected to have poor prognoses. The lower GNRI may be also considered as a marker of progression of valvular disease. In contrast, its changes have been reported in patients with pacemaker implantation and dialysis patients, where improvement in GNRI was associated with improved prognosis.^24^^,^^25^ The modifiability of GNRI is not fully clear, but if GNRI reflects nutritional status and inflammation, nutritional interventions, HF treatment, and disease management may be influential in increasing GNRI. Future study should clarify the modifiability of GNRI in TEER patients and its impact on prognosis.

### Study limitations

Several important limitations are inherent to the current registry data. First, this was a retrospective, single-Japanese, nonrandomized, unblinded, observational study, which might have introduced a selection bias, like previous cohort studies. Second, procedural and postprocedural echocardiographic parameters were not assessed in the central core laboratory. This may cause the risk of exposure misclassification including postprocedural MR grading or other important findings. However, we decided to use a consensus document based on the guidelines and share it with the participating institutions to minimize knowledge and technical gaps before registry enrollment. Additionally, several echocardiographic examinations for valvular heart disease had already been performed by experienced echocardiographers at the participating institutions when they started their TEER programs. Finally, the multivariable analysis did not capture potential undetected factors, although considerably important clinical variables were included. In addition, the proportional hazards assumption appears to be violated in the DMR group in this study, unlike in the overall and FMR groups, rendering the Cox proportional hazards results for DMR subject to further scrutiny. This limits the interpretation of the association between the GNRI and study outcomes. To elucidate the causal relationship between the GNRI and prognosis, it is necessary to clarify the modifiability of GNRI and to examine the impact of changes in GNRI due to interventions on prognosis.

## Conclusions

Our study aimed to demonstrate the value of the GNRI in predicting short- and long-term prognoses, separated by FMR and DMR, in patients undergoing TEER. In addition to being easy to measure, it is an excellent predictor of short- and long-term mortality risk with a single index, allowing accurate all-cause mortality risk stratification in patients from various backgrounds undergoing TEER. Although TEER is also effective for MR patients in malnourished states, the results of this study suggest that GNRI may be applicable to treatment prioritization in preprocedural assessment.

Perspectives **COMPETENCY IN PRACTICE-BASED LEARNING:** TEER is a minimally invasive treatment for patients with mitral regurgitation. Reports on the association between GNRI, a nutrition-related index, and mortality after TEER are limited; in the OCEAN-Mitral registry, low GNRI was associated not only with perioperative mortality, but also with long-term mortality. The GNRI, a simple index calculated from BMI and serum albumin, can be widely used to stratify patients at high risk of death in such a heterogeneous sample.

**TRANSLATIONAL OUTLOOK:** As the multicenter registry report of TEER, our data show that preprocedure GNRI values are useful as markers of all-cause mortality after treatment, regardless of the etiology of MR. To elucidate the causal relationship between the GNRI and prognosis, it is necessary to examine the impact of changes in the GNRI due to interventions on prognosis.

## Funding support and author disclosures

The OCEAN-Mitral registry, which is part of the Optimized Catheter Valvular Intervention–Structural Heart Disease (OCEAN-SHD) registry, was supported by Edwards Lifesciences, Medtronic Japan, Boston Scientific, Abbott Medical Japan, and the Daiichi-Sankyo Company. Drs Yamamoto, Kubo, Saji, Izumo, Watanabe, Nakajima, Ohno, Enta, Shirai, Mizuno, Boda, Kodama, and Amaki are clinical proctors of transcatheter edge-to-edge repair for Abbott Medical and have received lecture/consultant fees from Abbott Medical. Dr Asami has received speaker fees from Abbott Medical. Dr Yamaguchi is a clinical proctor of transcatheter edge-to-edge repair for Abbott Medical and has received a lecture fee and a scholarship donation from Abbott Medical. All other authors have reported that they have no relationships relevant to the contents of this paper to disclose.
