# Supplementary material for: Geriatric Nutritional Risk Index Assessment in Patients Undergoing Transcatheter Edge-to-Edge Repair
Source: JACC Adv. 2025 Feb 25;4(4):101631. doi: 10.1016/j.jacadv.2025.101631 (PMC11907445; doi:10.1016/j.jacadv.2025.101631)

**Supplemental Table 1. Baseline characteristics of study patients**

|  | Overall,  n=3554 | FMR,  n=2525 | DMR,  n=1029 | *P-value* |
| --- | --- | --- | --- | --- |
| Baseline clinical characteristics |  |  |  |  |
| Age, years | 78.7 ± 9.6 | 76.9 ± 9.7 | 83.0 ± 7.9 | < 0.001 |
| ≥ 80 years, n. (%) | 1981 (55.7%) | 1175 (46.5%) | 806 (78.3%) | < 0.001 |
| Male, n. (%) | 1951 (54.9%) | 1497 (59.3%) | 454 (44.1%) | < 0.001 |
| Height, cm | 156.9 ± 10.2 | 158.2 ± 9.9 | 153.8 ± 10.3 | < 0.001 |
| Weight, kg | 52.7 ± 11.5 | 53.8 ± 11.6 | 50.0 ± 10.9 | < 0.001 |
| BMI, kg/m^2^ | 21.3 ± 3.5 | 21.4 ± 3.5 | 21.0 ± 3.5 | 0.016 |
| Heart rate, bpm | 74.5 ± 15.6 | 74.5 ± 15.5 | 74.6 ± 15.9 | 0.825 |
| Clinical frailty scale, points | 4 (3–4) | 4 (3–4) | 4 (3–5) | < 0.001 |
| STS score for mitral valve replacement, (%, n=3350) | 8.9 (5.7–13.8) | 9.1 (5.7–14.2) | 8.6 (5.7–12.6) | 0.027 |
| Prior HFH within 12 months prior to enrollment, n. (%) | 2583 (73.0%) | 1913 (76.1%) | 670 (65.5%) | < 0.001 |
| NYHA class, III or IV | 2235 (62.9%) | 1631 (64.6%) | 604 (58.7%) | 0.001 |
| Preprocedural nutritional status and laboratory data |  |  |  |  |
| GNRI | 91.7 ± 9.6 | 91.6 ± 9.6 | 92.1 ± 9.6 | 0.173 |
| < 82, n. (%) | 590 (16.6%) | 423 (16.8%) | 167 (16.2%) | 0.228 |
| 82 to < 92, n. (%) | 1093 (30.8%) | 785 (31.1%) | 308 (29.9%) |  |
| 92–98, n. (%) | 849 (23.9%) | 616 (24.4%) | 233 (22.7%) |  |
| ≥ 98, n. (%) | 1022 (28.7%) | 701 (27.7%) | 321 (31.2%) |  |
| Albumin, g/dl | 3.6 ± 0.6 | 3.6 ± 0.6 | 3.7 ± 0.5 | 0.004 |
| BNP, (pg/ml, n=2579) | 351.1 (169.9–711.0) | 431.6 (213.5–837.9) | 228.4 (113.6–437.0) | < 0.001 |
| Estimated glomerular filtration rate, mL/min/1.73 m^2^ | 39.2 ± 19.4 | 37.6 ± 19.4 | 42.9 ± 18.8 | < 0.001 |
| < 30 mL/min/1.73 m^2^, n. (%) | 1181 (33.4%) | 908 (36.2%) | 273 (26.6%) | < 0.001 |
| Hemoglobin, g/dL | 11.7 ± 1.9 | 11.7 ± 1.9 | 11.7 ± 1.7 | 0.241 |
| High-sensitivity CRP, (mg/dL, n=3325) | 0.18 (0.07–0.60) | 0.19 (0.07–0.68) | 0.13 (0.06–0.47) | < 0.001 |
| Comorbidities |  |  |  |  |
| Hypertension, n. (%) | 2318 (65.2%) | 1598 (63.3%) | 720 (70.0%) | < 0.001 |
| Dyslipidemia, n. (%) | 1724 (48.5%) | 1331 (52.7%) | 393 (38.2%) | < 0.001 |
| Diabetes mellitus, n. (%) | 941 (26.5%) | 795 (31.5%) | 146 (14.2%) | < 0.001 |
| Chronic kidney disease, n. (%) | 3080 (86.7%) | 2218 (87.8%) | 862 (83.8%) | 0.002 |
| Dialysis dependent, n. (%) | 215 (6.0%) | 184 (7.3%) | 31 (3.0%) | < 0.001 |
| Atrial fibrillation / flutter, n. (%) | 2184 (61.5%) | 1597 (63.2%) | 587 (57.0%) | 0.001 |
| Prior stroke, n. (%) | 409 (11.5%) | 289 (11.4%) | 120 (11.7%) | 0.862 |
| Liver cirrhosis, n. (%) | 59 (1.7%) | 44 (1.7%) | 15 (1.5%) | 0.664 |
| Chronic obstructive lung disease, n. (%) | 301 (8.5%) | 214 (8.5%) | 87 (8.5%) | 1.0 |
| Peripheral artery disease, n. (%) | 348 (9.8%) | 267 (10.6%) | 81 (7.9%) | 0.015 |
| Echocardiographic data |  |  |  |  |
| LV Ejection fraction, % | 44.8 ± 16.5 | 38.6 ± 14.1 | 60.0 ± 11.4 | < 0.001 |
| < 40%, n. (%) | 1612 (45.4%) | 1529 (60.6%) | 83 (8.1%) | < 0.001 |
| LV end-diastolic diameter, mm | 56.6 ± 10.4 | 59.0 ± 10.3 | 50.7 ± 7.7 | < 0.001 |
| LV end-systolic diameter, mm | 44.0 ± 13.5 | 48.3 ± 12.8 | 33.6 ± 8.6 | < 0.001 |
| LA volume index, cm^3^/m^2^ | 86.4 ± 50.1 | 86.9 ± 52.0 | 85.2 ± 45.3 | 0.354 |
| MR characteristics |  |  |  |  |
| Moderate-severe MR, n. (%) | 3497 (98.4%) | 2475 (98.0%) | 1022 (99.3%) | 0.005 |
| MR PISA EROA, cm^2^ | 0.38 ± 0.20 | 0.34 ± 0.16 | 0.48 ± 0.23 | < 0.001 |
| MR regurgitation volume, ml | 55.4 ± 30.4 | 49.9 ± 27.4 | 69.0 ± 33.1 | < 0.001 |
| TMPG, mmHg | 1.87 ± 1.09 | 1.73 ± 0.99 | 2.19 ± 1.25 | < 0.001 |

Values are numbers (%) or mean ± SD, median with interquartile range. BMI, body mass index; STS score, Society of Thoracic Surgeons Predictive Risk of Mortality; HFH, heart failure hospitalization; Society of Thoracic Surgeons Predictive Risk of Mortality; NYHA, New York Heart Association; BNP, B-type natriuretic peptide; CRP, C-reactive protein; LV, left ventricular; MR, mitral regurgitation: PISA, proximal isovelocity surface area; EROA, effective regurgitant orifice area; TMPG, transmitral mean pressure gradient.

**Supplemental Table 2. Univariable and multivariable Cox regression analyses for cardiac and non-cardiac death**

1. **Cardiac death**

|  | Overall | | | FMR | | | DMR | | |
| --- | --- | --- | --- | --- | --- | --- | --- | --- | --- |
| **Univariable analysis** | HR | 95% CI | p-value | HR | 95% CI | p-value | HR | 95% CI | p-value |
| GNRI >98 | Reference |  |  | Reference |  |  | Reference |  |  |
| GNRI 92 to ≤ 98 | 1.62 | 1.19–2.19 | 0.002 | 1.37 | 0.97–1.94 | 0.072 | 2.90 | 1.44–5.83 | 0.003 |
| GNRI 82 to < 92 | 2.74 | 2.09–3.59 | <0.001 | 2.70 | 2.00–3.64 | <0.001 | 3.07 | 1.57–5.99 | 0.001 |
| GNRI < 82 | 5.33 | 4.01–7.08 | <0.001 | 4.95 | 3.61–6.78 | <0.001 | 7.52 | 3.82–14.82 | <0.001 |
| **Multivariable analysis** |  |  |  |  |  |  |  |  |  |
| GNRI >98 | Reference |  |  | Reference |  |  | Reference |  |  |
| GNRI 92 to ≤ 98 | 1.13 | 0.77–1.67 | 0.53 | 0.91 | 0.59–1.40 | 0.67 | 3.59 | 1.35–9.58 | 0.011 |
| GNRI 82 to < 92 | 1.75 | 1.24–2.48 | 0.002 | 1.48 | 1.01–2.15 | 0.043 | 4.48 | 1.70–11.82 | 0.003 |
| GNRI < 82 | 2.65 | 1.79–3.94 | <0.001 | 2.09 | 1.36–3.21 | <0.001 | 10.10 | 3.50–29.13 | <0.001 |

1. **Non-cardiac death**

|  | Overall | | | FMR | | | DMR | | |
| --- | --- | --- | --- | --- | --- | --- | --- | --- | --- |
| **Univariable analysis** | HR | 95% CI | p-value | HR | 95% CI | p-value | HR | 95% CI | p-value |
| GNRI >98 | Reference |  |  | Reference |  |  | Reference |  |  |
| GNRI 92 to ≤ 98 | 2.04 | 1.41–2.94 | <0.001 | 1.78 | 1.17–2.72 | 0.008 | 2.95 | 1.38–6.31 | 0.005 |
| GNRI 82 to < 92 | 3.35 | 2.40–4.68 | <0.001 | 3.33 | 2.28–4.86 | <0.001 | 3.31 | 1.60–6.82 | 0.001 |
| GNRI < 82 | 5.52 | 3.87–7.89 | <0.001 | 5.27 | 3.51–7.90 | <0.001 | 6.37 | 3.00–13.50 | <0.001 |
| **Multivariable analysis** |  |  |  |  |  |  |  |  |  |
| GNRI >98 | Reference |  |  | Reference |  |  | Reference |  |  |
| GNRI 92 to ≤ 98 | 1.40 | 0.88–2.23 | 0.15 | 1.22 | 0.72–2.09 | 0.46 | 2.36 | 0.89–6.25 | 0.084 |
| GNRI 82 to < 92 | 2.10 | 1.36–3.24 | <0.001 | 2.00 | 1.22–3.29 | 0.006 | 2.52 | 1.00–6.32 | 0.049 |
| GNRI < 82 | 2.57 | 1.56–4.22 | <0.001 | 2.47 | 1.41–4.33 | 0.002 | 3.43 | 1.05–9.50 | 0.041 |

Multivariable models were adjusted for: age, sex, hemoglobin, prior HFH within 12 months prior to enrollment, New York Heart Association functional class III or IV, Heart rate, atrial fibrillation/flutter, eGFR, clinical frailty scale, LVEF < 40%, Diabetes mellitus, BNP, Dialysis dependent, Peripheral artery disease, Functional MR (Overall only)

CI, confidence interval; eGFR, estimated glomerular filtration rate; HFH, heart failure hospitalization; HR, hazard ratio; LVEF, left ventricle ejection fraction.

**Supplemental Table 3. Net reclassification improvement and integrated discrimination improvement for comparison among basic components**

|  | NRI | 95% CI | p-value | IDI | 95% CI | p-value |
| --- | --- | --- | --- | --- | --- | --- |
| Clinical model | Reference |  |  | Reference |  |  |
| Clinical model + GNRI | 0.292 | 0.198 to 0.385 | <0.001 | 0.017 | 0.011 to 0.023 | <0.001 |
| Clinical model + Albumin | 0.130 | 0.036 to 0.224 | 0.007 | 0.009 | 0.005 to 0.013 | <0.001 |
| Clinical model + body height | -0.020 | -0.114 to 0.075 | 0.684 | 0 | -0.0001 to 0.0001 | 0.649 |
| Clinical model + body weight | 0.231 | 0.138 to 0.324 | <0.001 | 0.007 | 0.003 to 0.011 | <0.001 |
| Clinical model + BMI < 25 | 0.020 | -0.045 to 0.085 | 0.537 | 0.0005 | -0.001 to 0.002 | 0.378 |
| Clinical model + Albumin | Reference |  |  | Reference |  |  |
| Clinical model + GNRI | 0.254 | 0.160 to 0.347 | <0.001 | 0.008 | 0.005 to 0.011 | <0.001 |
| Clinical model + body height | Reference |  |  | Reference |  |  |
| Clinical model + GNRI | 0.287 | 0.193 to 0.380 | <0.001 | 0.017 | 0.011 to 0.023 | <0.001 |
| Clinical model + body weight | Reference |  |  | Reference |  |  |
| Clinical model + GNRI | 0.090 | -0.004 to 0.189 | 0.06 | 0.010 | 0.004 to 0.016 | <0.001 |
| Clinical model + BMI < 25 | Reference |  |  | Reference |  |  |
| Clinical model + GNRI | 0.291 | 0.198 to 0.385 | <0.001 | 0.017 | 0.011 to 0.023 | <0.001 |

Clinical model: age, sex, hemoglobin, prior HFH within 12 months prior to enrollment, New York Heart Association functional class III or IV, Heart rate, atrial fibrillation/flutter, eGFR, LVEF < 40%, Diabetes mellitus, BNP, Dialysis dependent, Peripheral artery disease, Functional MR

BMI, body mass index; CI, confidence interval; IDI, integrated discrimination improvement; NRI, net reclassification improvement; LVEF, left ventricle ejection fraction.

Supplemental Figure 1. Kaplan–Meier all-cause mortality and competing risk for HFH according to GNRI, stratified by BMI of 20 kg/m^2^ and 25 kg/m^2^.

Kaplan–Meier curve of all-cause mortality and competing risk for HFH in the cohort stratified BMI of 20 kg/m^2^ and 25 kg/m^2^.

BMI < 20 kg/m^2^ (A), BMI 20–25 kg/m^2^ (B), BMI ≥ 25 kg/m^2^ (C).


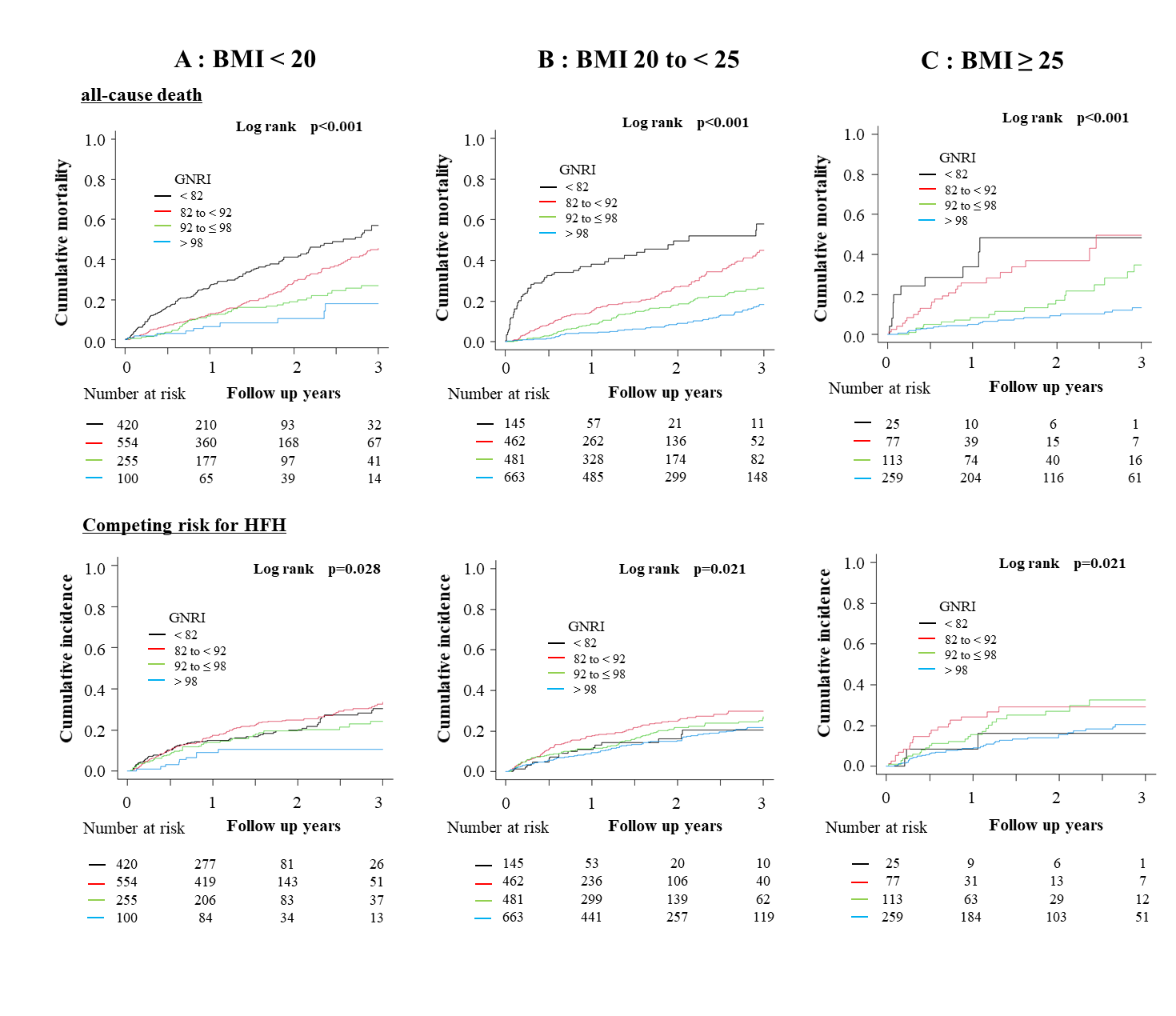


**Supplemental Figure 2.** Kaplan–Meier curve of all-cause mortality and competing risk for HFH according to GNRI, stratified by median BNP.

Kaplan–Meier curve of all-cause mortality and competing risk for HFH overall, stratified by median BNP. BNP < median (A), BNP ≥ median (B).

BNP, B-type natriuretic peptide; HFH, heart failure hospitalization


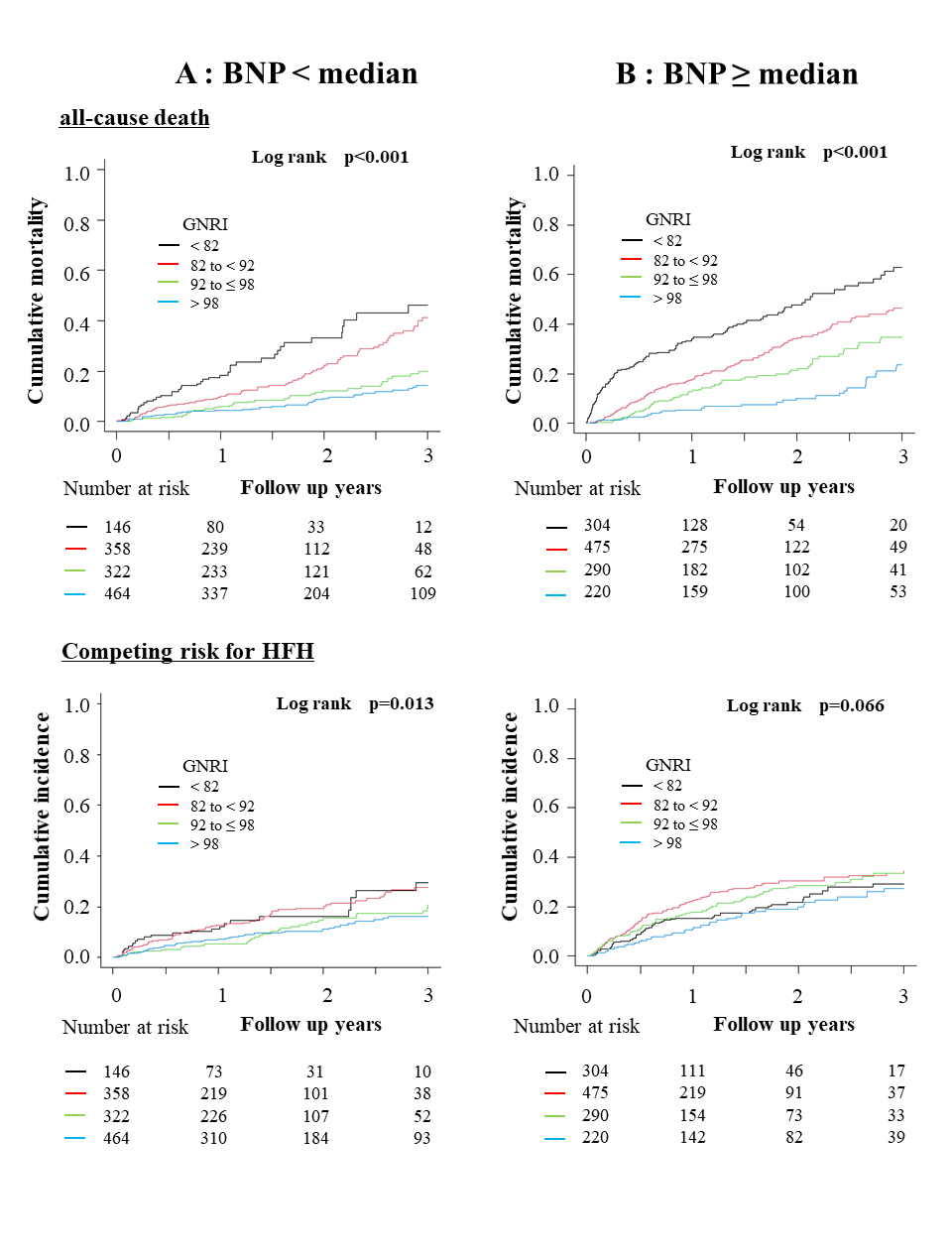

Supplement: Supplemental Tables 1, 2, and 3, and Supplemental Figures 1 and 2 [file mmc1.docx]
